# Brassinosteroids Alleviate Salt Stress by Enhancing Sugar and Glycine Betaine in Pepper (*Capsicum annuum* L.)

**DOI:** 10.3390/plants13213029

**Published:** 2024-10-29

**Authors:** Yujie Jin, Ping Yang, Jian Li, Yongchao Yang, Ruopeng Yang, Hongbo Fu, Jie Li

**Affiliations:** 1 College of Biological and Agricultural Sciences, Honghe University, Mengzi 661100, China; jinyujie2024@163.com (Y.J.);; 2 College of Horticulture and Forestry, Tarim University, Alar 843300, China; 3 College of Horticulture, Gansu Agriculture University, Lanzhou 730070, China; 4 College of Chemistry and Resources Engineering, Honghe University, Mengzi 661100, China

**Keywords:** pepper, salt stress, brassinosteroids, enhancing sugar, glycine betaine

## Abstract

Salt stress is a major abiotic factor that negatively impacts the growth, performance, and secondary metabolite production in pepper (*Capsicum annuum* L.) plants. Brassinosteroids (BRs) play a crucial role in enhancing plant tolerance to abiotic stress, yet their potential in mitigating salt stress in pepper plants, particularly by promoting sugar and glycine betaine accumulation, remains underexplored. In this study, we investigated the effects of the foliar application of 2,4-epibrassinolide (EBR) on salt-stressed pepper seedlings. Our findings revealed that EBR treatment significantly increased the levels of proline, sugar, and glycine betaine under salt stress compared to untreated controls. Moreover, EBR enhanced the antioxidant defense mechanisms in pepper seedlings by increasing sugar and glycine betaine levels, which contributed to the reduction of reactive oxygen species (ROS) and malondialdehyde (MDA) accumulation.

## 1. Introduction

Salinity, heat, cold, and drought are among the most harmful abiotic stresses, adversely impacting plant growth, biomass accumulation, fruit quality, and yield in various ways [1]. According to the Food and Agriculture Organization of the United Nations (FAO), over 10% of global agricultural land is currently affected by salt stress, posing a significant threat to food security [2]. Plants exposed to salt stress undergo a series of physiological and biochemical changes that inhibit their normal functions. High salt concentrations increase the osmotic pressure within plant cells, causing water loss and cellular dehydration, leading to osmotic stress. Additionally, elevated salt levels reduce photosynthesis efficiency, limiting plant productivity [3], and thereby disrupting regular physiological activities.

Soils affected by salinity contain diverse ions, primarily Na^+^ and Cl^−^. These ions accumulate in plant tissues, disrupting the uptake of essential nutrients such as Ca^+^ and causing ionic imbalances. This results in ionic toxicity and oxidative damage to plants [4,5,6]. Given the growing impact of salt stress, it is critical to address these challenges to maintain plant yield and quality.

Sugars are an indispensable material basis for plant life’s activities. They provide energy and play a crucial role in maintaining cell structure stability, regulating growth and development, and coping with adversity and stress [7]. Numerous studies have provided substantial evidence that sugars play a pivotal role in enhancing plant tolerance to a diverse array of abiotic stresses, including salt stress [8]. The mechanisms of action are complex and multifaceted, primarily supporting plant adaptation and resistance to stress [9]. The presence of salt in the soil, or salt stress, increases the concentration of salt ions. This, in turn, causes a reduction in the water potential of plant cells, which subsequently leads to water loss and affects normal plant growth. The accumulation of soluble sugars by plants enables an increase in the cytosol concentration, an elevation in the osmotic pressure of the cell membrane, and a reduction in the water potential of the cell. This process serves to alleviate water loss due to salt stress [10]. The accumulation of reactive oxygen species in plant cells resulting from salt stress has been demonstrated to trigger oxidative stress, thereby causing damage to the cell structure and function. It has been demonstrated that sugars can engage in the antioxidant defense system of plants via several pathways, thereby mitigating the oxidative damage caused by salt stress. Primarily, sugars can function as scavengers of reactive oxygen species, directly reacting with these species and thereby reducing their toxicity. Secondly, sugars can act as signaling molecules, inducing the expression of antioxidant enzymes, including superoxide dismutase (SOD), catalase (CAT), and peroxidase (POD), thereby enhancing the antioxidant capacity of plants [11]. In addition, sugars regulate protein synthesis by influencing gene expression and metabolic pathways [12]. Overall, sugars play a multifaceted role in the salt stress response by regulating osmotic pressure, antioxidants, membrane and protein protection, and gene expression [13,14].

Glycine betaine (GB) also plays a crucial role in plant responses to salt stress. As an effective osmoprotectant, GB accumulates in plants subjected to salt stress, thereby increasing the intracellular osmotic pressure and maintaining cellular water balance. By elevating the cytosol concentration and reducing the cell water potential, GB can mitigate the detrimental effects of salt stress on plant cells, ensuring the maintenance of normal physiological functions [15]. Furthermore, GB can bind to cell membrane lipids, thereby enhancing membrane stability and improving membrane salt resistance. By interacting with membrane lipids, GB can reduce membrane fluidity, lower the phase transition temperature of membrane lipids, and improve membrane stability, thereby reducing membrane damage caused by salt stress. GB is a potent antioxidant that scavenges ROS and protects cells from oxidative damage. GB can reduce the toxicity of ROS by directly reacting with them and by inducing the expression of antioxidant enzymes in plants [16,17].

Brassinosteroids (BRs) are highly active plant steroid hormones [18,19]. Extensive research shows that BRs have significant physiological effects on plants, particularly in enhancing tolerance to various abiotic stresses, including salt stress [20,21,22,23]. BRs regulate key physiological processes, such as stomatal conductance, photosynthesis, and ion uptake, while also activating antioxidant defense systems to mitigate oxidative damage caused by salt stress [24,25].

Pepper (*Capsicum annuum* L.) is an economically important vegetable crop worldwide. Salt stress negatively affects seed germination, plant development, and fruit maturation in pepper plants. Previous studies have shown that BRs’ application can enhance the salt tolerance in various plants, including *Arabidopsis* [24], *Oryza sativa* [26], and *Solanum lycopersicum* [27]. Despite extensive research on salt stress, the role of BRs in enhancing the salt tolerance in pepper, particularly in relation to sugar and glycine betaine accumulation, remains underexplored. Further investigation is needed to clarify the mechanisms by which BRs mediate salt stress tolerance through the regulation of sugars and glycine betaine in pepper plants.

## 2. Materials and Methods

### 2.1. Plant Materials and Treatment

The “169” pepper variety, provided by the Beijing Academy of Agriculture and Forestry Sciences, was used as the experimental material. Pepper seeds were selected, disinfected, soaked, and placed in Petri dishes containing filter paper or cotton. The seeds were then incubated in an artificial climate chamber at 24–26 °C, with distilled water sprayed until germination occurred. Subsequently, the seedlings were transplanted into trays containing a substrate mixture of coconut coir, vermiculite, and perlite in a 3:1:1 (*v*/*v*) ratio. Once the seedlings developed 4–5 true leaves, they were transferred to planting pots (10 cm diameter) and placed in a growth chamber set to the following conditions: light intensity of 200 μmol/m^2^·s, a 12 h photoperiod, diurnal temperatures of 28/18 °C (day/night), relative humidity of 65%, and a CO_2_ concentration of 200 μmol/mol. The seedlings were allowed to acclimate for 7 days.

After acclimation, a watering regimen was initiated using half-strength Hoagland’s nutrient solution supplemented with 200 mmol NaCl to induce salt stress. Additionally, different concentrations of 2,4-epibrassinolide (EBR (0 mol/L, 10^−5^ mol/L, 10^−6^ mol/L, 10^−7^ mol/L, 10^−8^ mol/L, 10^−9^ mol/L) were foliar-sprayed every 2 days. One week after treatment initiation, phenotypic analyses were conducted to determine the optimal EBR concentration for enhancing salt stress tolerance in pepper seedlings.

Based on the concentration screening results, a 10^−7^ mol/L EBR solution was selected for further experiments. The experimental design included four treatments: Control (spraying with distilled water and watering with half-strength Hoagland’s nutrient solution), Control + EBR (spraying with 10^−7^ mol/L EBR and watering with half-strength Hoagland’s nutrient solution), NaCl (spraying with distilled water and watering with half-strength Hoagland’s nutrient solution supplemented with 200 mmol/L NaCl), and NaCl + EBR (spraying with 10^−7^ mol/L EBR and watering with half-strength Hoagland’s nutrient solution supplemented with 200 mmol/L NaCl). Each treatment was replicated three times. The Control + EBR and NaCl + EBR groups received daily sprays of 10^−7^ mol/L EBR until water droplets dripped from the leaf surfaces. All treatments received equal volumes of half-strength Hoagland’s nutrient solution. Pepper seedlings were harvested 7 days after the start of treatments.

### 2.2. Leaf Microstructure Analysis

Leaf microstructure was analyzed following the method of Anderson et al. [28]. Fresh leaves were attached to double-sided adhesive tape and mounted on metal stubs for scanning electron microscopy (SEM) analysis. The abaxial surfaces of the leaves were examined using a Hitachi S-3400N SEM (Hitachi, Tokyo, Japan) at an accelerating voltage of 3.0 kV and a pressure of 60 Pa.

### 2.3. Photosynthetic Parameters and Chlorophyll Fluorescence Analysis

Photosynthetic parameters and chlorophyll fluorescence were measured according to Li et al. [29]. Gas exchange parameters, including net photosynthetic rate (Anet), stomatal conductance (g_s_), internal CO_2_ concentration (Ci), and transpiration rate (Tr), were assessed using a portable photosynthetic system (Li-COR 6400, Li-COR, Lincoln, NE, USA) on the third fully expanded leaf. Chlorophyll fluorescence parameters were determined using a leaf chamber fluorometer (Li-COR 6400-40, Li-COR, Lincoln, NE, USA).

### 2.4. Electrolyte Leakage

Electrolyte leakage was quantified using the method described by Sullivan and Ross (1979) [30]. Twenty leaf discs were placed in boiling tubes containing 10 mL of deionized water, and the electrical conductivity was measured (EC_a_). The solutions were then heated at 45 °C and 55 °C for 30 min each, and electrical conductivity was measured again (EC_b_). Subsequently, the solutions were boiled at 100 °C for an additional 10 min, and electrical conductivity was recorded (EC_c_). Electrolyte leakage (%) was calculated using the formula: Electrolyte leakage (%) = EC_b_ − EC_a_/EC_c_ × 100.

### 2.5. Free Radical Production and Lipid Peroxidation

The malondialdehyde (MDA) concentration was determined following Heath and Packer (1968) [31]. MDA levels were calculated from the absorbance at 532 nm, with nonspecific turbidity at 600 nm subtracted. The superoxide production rate was measured using a modified method of Elstner and Heupel (1976) [32], with measurements taken at 530 nm. The concentration of O_2_^−^ was determined using a standard curve of NaNO_2_.

The hydrogen peroxide (H_2_O_2_) content was estimated according to Patterson et al. (1984) [33], based on the absorbance change of the titanium peroxide complex at 415 nm. The histochemical localization of H_2_O_2_ and O_2_^−^ in the leaves was performed using nitroblue tetrazolium (NBT) and 3,3-diaminobenzidine (DAB) staining, respectively, following the protocol of Khokon et al. [34].

### 2.6. Enzyme Assays

A quantity of 0.3 g of pepper leaf tissue was placed in a solution of 2 mL of PBS buffer (containing 0.2 mol EDTA and 50 mmol 2% PVP (*w*/*v*), pH 7.8) and homogenized in an ice-water bath. The homogenate was centrifuged at 12,000× *g* for 20 min at 4 °C. The supernatant was collected for enzyme assays using a UV-2410 spectrophotometer. Superoxide Dismutase (SOD) activity was determined by its ability to inhibit the reduction of nitroblue tetrazolium (NBT). Absorbance was monitored at 560 nm. One unit of SOD was defined as the amount of extract causing a 50% inhibition of the NBT reduction rate [35]. Peroxidase (POD) activity was quantified by measuring the characteristic absorption peak at 460 nm of tetra-o-methoxyphenol generated from guaiacol [36]. Catalase (CAT) activity was measured by monitoring the decomposition of hydrogen peroxide in the presence of potassium permanganate. Absorbance was recorded at 240 nm. One unit of CAT was defined as the amount of H_2_O_2_ (µmol) decomposed per minute [37]. Ascorbate Peroxidase (APX) activity was determined by monitoring the rate of ascorbate oxidation at 290 nm [38]. Enzyme activities were expressed as units per milligram of protein.

### 2.7. Ascorbate and Glutathione Assays

Ascorbate (AsA) and Dehydroascorbate (DHA). Total ascorbate was determined following Costa et al. (2002) [39], with slight modifications. A sample of 0.3 g of roots were homogenized in ice-cold 5% (*w*/*v*) trichloroacetic acid (TCA) and centrifuged at 12,000× *g* for 10 min. Half of the sample was assayed for total ascorbate (AsA + DHA) and the other half for AsA only. The DHA concentration was calculated by subtracting AsA from the total ascorbate. Absorbance was measured at 525 nm.

Glutathione (GSH and GSSG). Glutathione levels were measured according to Baker et al. (1990) [40], with minor modifications. Non-protein thiols were extracted by homogenizing 0.2 g of roots in 1.6 mL of 5% sulfosalicylic acid and centrifuged at 14,000× *g* for 10 min. The reaction mixture included 0.2 mL of 6 mmol 5,5′-dithiobis (2-nitrobenzoic acid) (DTNB), 0.1 mL of 2 mmol NADPH, 0.5 mL of 0.1 mol KHPO_4_ buffer with 5 mmol EDTA (pH 8.0), 1 U of glutathione reductase, and 0.1 mL of enzyme extract. Changes in absorbance were recorded at 412 nm using a spectrophotometer (UV-2450, Shimadzu, Japan). GSH was calculated by subtracting GSSG from the total glutathione.

Concentrations of AsA, DHA, GSH, and GSSG were expressed on a fresh weight basis.

### 2.8. Proline Accumulation Determination

The proline content was measured using the method of Bates et al. [41]. Fresh leaf samples were extracted with sulfosalicylic acid. An equal volume of glacial acetic acid and ninhydrin solution was added to the extract. The samples were heated at 100 °C, followed by the addition of 5 mL toluene. The absorbance of the toluene layer was measured at 528 nm using a spectrophotometer.

### 2.9. Sugar Content Determination

Soluble sugars, including sucrose and fructose, were quantified following Buysse and Merckx (1993) [42], using glucose as a standard. Additionally, the glucose content was measured according to Jones et al. (1977) [43]. Approximately 2 g of fresh material was placed in a 25 mL tube containing 10 mL distilled water and boiled at 100 °C for 10 min. After cooling to room temperature, samples were filtered into clean 25 mL volumetric flasks and diluted with distilled water to volume. Subsequently, 1 mL of the extract was mixed with 5 mL anthrone reagent and boiled at 100 °C for 10 min. Absorbance was measured at 625 nm, and the soluble sugar concentration was calculated per gram of fresh weight based on the glucose standard curve.

### 2.10. Na^+^ and K^+^ Content Determination

Samples were dried at 75 °C in a drying oven until powdered. A 50 mg sample was treated in a furnace and weighed accurately. The sample was then dissolved in concentrated acid (HNO_3_: perchloric acid, 3:1 *v*/*v*) at 250 °C and diluted to 100 mL with distilled water. Sodium and potassium concentrations were determined using inductively coupled plasma mass spectrometry (ICP-MS).

### 2.11. Abscisic Acid (ABA) Determination

ABA was extracted and analyzed according to the method of Dobrev et al. [44]. The whole process consisted of crushing the tissue in liquid nitrogen, extracting it with 80% methanol at low temperatures, and then purifying it through several steps such as decolorization, pH adjustment, and ethyl acetate extraction. Finally, the extracted ABA was analyzed by high-performance liquid chromatography (HPLC) on a C18 column with methanol and 0.2% acetic acid as mobile phases. The absorbance was measured at 260 nm.

### 2.12. Glycine Betaine (GB) Assays

The content of GB was determined using Reinecke’s method with slight variations in his method [45]. The experiments consisted of preparing a standard solution of betaine, extracting glycine betaine from pepper leaves using a methanol–chloroform mixture, and measuring the absorbance of the extracted betaine at 525 nm using a spectrophotometer. A standard curve was also used to quantify the betaine content in pepper leaves.

### 2.13. Total RNA Extraction and Gene Expression Analysis

Total RNA was extracted from pepper leaves using the Trizol reagent following the manufacturer’s instructions. Residual DNA was removed using a purification column. Relative gene expression levels were calculated using the 2^−ΔΔCt^ method. The primer sequences used for quantitative reverse transcription polymerase chain reaction (qRT-PCR) amplification are listed in Table S2.

### 2.14. Data Statistics and Analysis

Data were compiled using Microsoft Office Excel 2019 and processed using Origin 2021 software. Statistical analyses of the indicators were performed, and corresponding figures (e.g., bar charts) were generated. All figures included in the manuscript were organized using Adobe Illustrator 2023.

## 3. Results

### 3.1. Foliar Application of 10^−7^ mol/L EBR Enhances the Resilience of Pepper Seedlings Against Salt-Induced Stress

**Figure 1:**
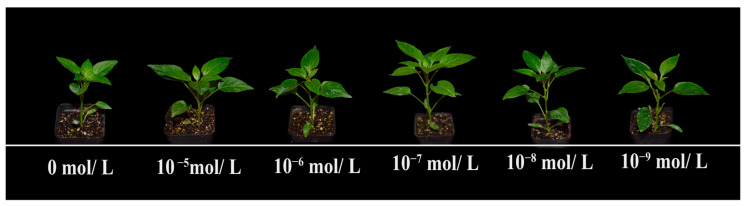
Morphology of pepper seedlings under different concentrations of EBR. Treatments from left to right: 0 mol/L, 10^−5^ mol/L, 10^−6^ mol/L, 10^−^⁷ mol/L, 10^−^⁸ mol/L, and 10^−^⁹ mol/L EBR added to the half-strength Hoagland nutrient solution and 200 mmol/L NaCl solution.

**Figure 2:**
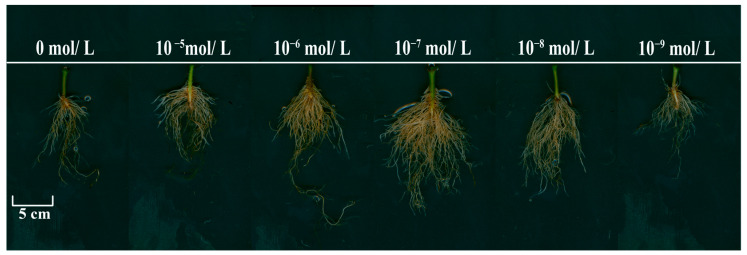
Root morphology of pepper seedlings under different EBR concentrations.

This study investigated the effects of foliar spraying with six different concentrations of EBR on pepper seedlings subjected to NaCl-induced salt stress. The goal was to determine the optimal EBR concentration for alleviating the negative effects of salt stress. Applying 200 mmol/L NaCl for seven days significantly reduced the vigor of six-leaf pepper seedlings compared to non-saline conditions. Key variables, such as phenotypic changes in the aboveground and underground parts (e.g., stem diameter, plant height, fresh weight, dry weight, and leaf area), were examined (see Supplementary Table S1, Figure 1 and Figure 2). The results showed that EBR sprays significantly alleviated salt-induced damage, with the 10^−7^ mol/L EBR concentration yielding the most pronounced improvements in plant height, stem diameter, fresh and dry weight, and leaf area compared to other treatments (Figure 1 and Supplementary Table S1).

### 3.2. EBR Mitigates Photosystem Damage and Improves Chlorophyll Fluorescence in Pepper Seedlings Under Salt Stress

**Figure 3:**
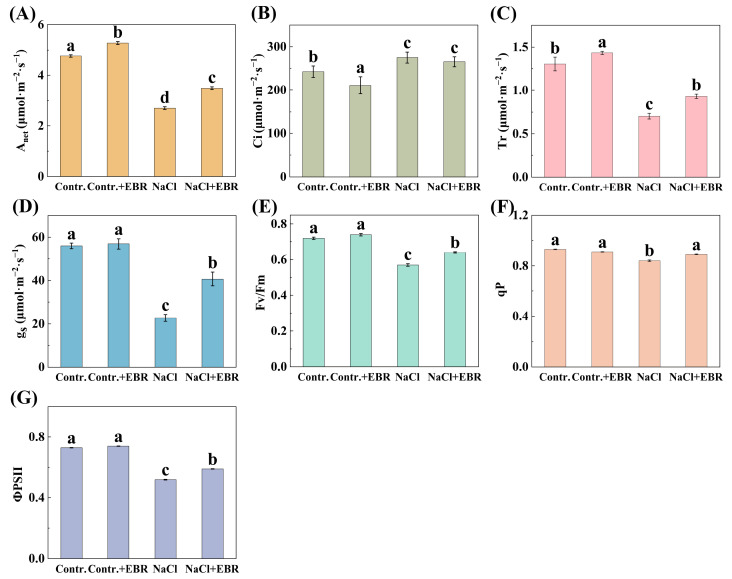
Changes in photosynthetic and chlorophyll fluorescence parameters under different treatments. Treatments include control (Contr.), EBR alone (Contr. + EBR), NaCl stress (NaCl), and EBR with NaCl stress (NaCl + EBR). (**A**) Changes in Anet. (**B**) Changes in Ci. (**C**) Changes in Tr. (**D**) Changes in g_s_. (**E**) Changes in Fv/Fm. (**F**) Changes in qP. (**G**) Changes in ΦPSII. Significant differences are indicated by different letters (*p* < 0.05, one-way ANOVA).

Salt stress significantly inhibited the net photosynthetic rate, intercellular CO_2_ levels, transpiration rate, and stomatal conductance in pepper leaves (Figure 3, *p* < 0.05). However, the foliar application of 10^−7^ mol/L EBR under salt stress significantly restored these parameters, leading to a 632.79% increase in photosynthesis, a 24.92% rise in intercellular CO_2_, a 656.27% increase in the transpiration rate, and a 130.82% improvement in stomatal conductance compared to NaCl stress alone. Chlorophyll fluorescence parameters (Fv/Fm, qP, and ΦPSII) were also improved by EBR application under salt stress, suggesting EBR’s protective role in maintaining photosynthetic efficiency.

### 3.3. EBR Reduces Oxidative Stress by Modulating ROS in Salt-Stressed Pepper Seedlings

**Figure 4:**
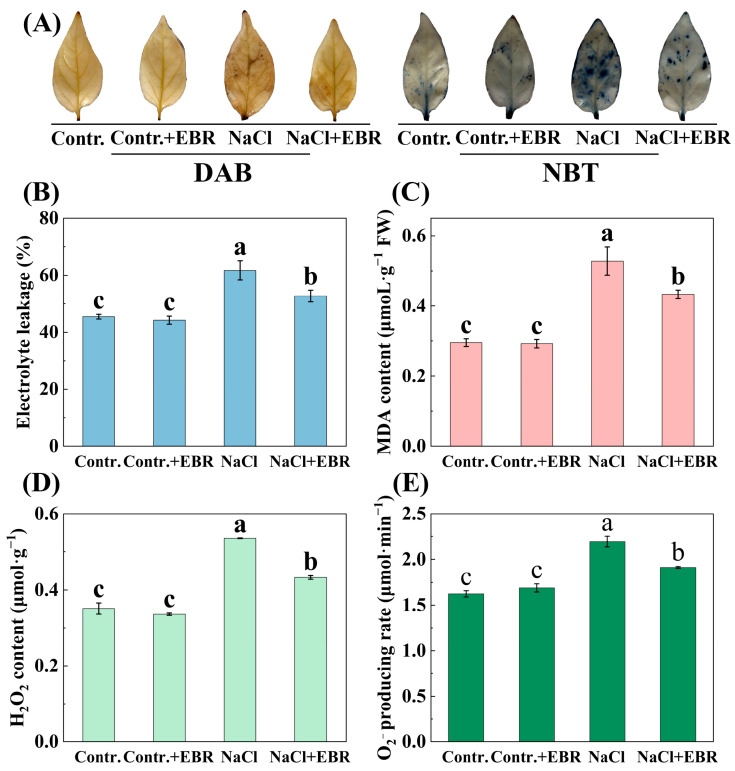
ROS content and staining (DAB and NBT) in pepper seedling leaves under different treatments. (**A**) DAB and NBT staining of pepper leaves. (**B**) Changes in Electrolyte leakage. (**C**) Changes in MDA content. (**D**) Changes in H_2_O_2_ content. (**E**) Changes in O_2_^−^ content. Different lowercase letters in the Figure indicate significant differences (*p* < 0.05, one-way ANOVA).

NaCl stress significantly increased electrolyte leakage, the malondialdehyde (MDA) content, and ROS (H_2_O_2_ and O_2_^−^) levels in pepper seedlings (Figure 4, *p* < 0.05). Exogenous EBR treatment under salt stress reduced these oxidative stress markers. For example, electrolyte leakage and the MDA content decreased by 14.63% and 18.02%, respectively, when compared to NaCl stress alone. Furthermore, EBR was observed to reduce the levels of H_2_O_2_ and O_2_^−^ by 19.07% and 12.75%, respectively. This was accompanied by a notable decrease in the intensity and visibility of DAB and NBT staining on pepper leaves (Figure 4A), which provides additional evidence of its role in mitigating salt-induced oxidative damage.

### 3.4. EBR Enhances Antioxidant Defense Mechanisms in Salt-Stressed Pepper Seedlings

**Figure 5:**
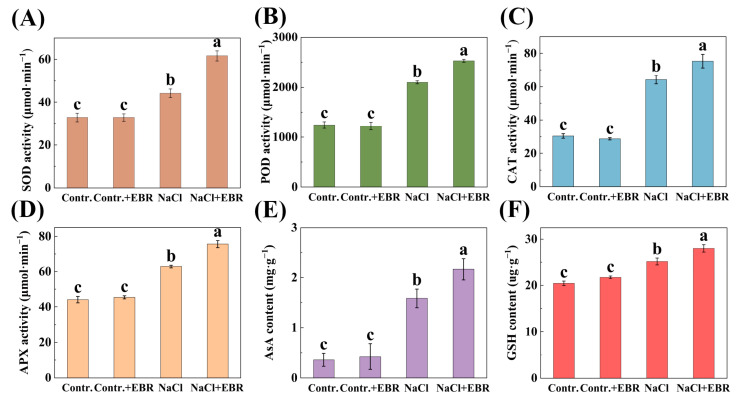
Changes in the antioxidant system of pepper seedlings under different treatments. (**A**) Changes in SOD content. (**B**) Changes in POD content. (**C**) Changes in CAT content. (**D**) Changes in APX content. (**E**) Changes in AsA content. (**F**) Changes in GSH content. Different lowercase letters in the Figure indicate significant differences (*p* < 0.05, one-way ANOVA).

Salt stress significantly elevated the activities of antioxidant enzymes (SOD, POD, CAT, and APX) and the levels of antioxidants (AsA and GSH) in pepper leaves. However, EBR application under salt stress further boosted these antioxidant parameters (Figure 5). This suggests that EBR enhances the antioxidant defense system, helping to alleviate oxidative damage induced by salt stress.

### 3.5. EBR Modulates Sugar and Proline Accumulation Under Salt Stress

**Figure 6:**
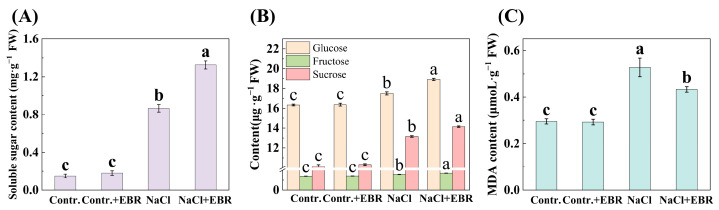
Changes in sugars and proline content under different treatments. (**A**) Changes in Soluble sugar content. (**B**) Changes in Glucose, Fructose, and Sucrose content. Orange for Glucose, green for Fructose, and red for Sucrose. (**C**) Changes in proline content. Different lowercase letters in the Figure indicate significant differences (*p* < 0.05, one-way ANOVA).

Salt stress significantly increased soluble sugar, glucose, fructose, sucrose, and proline levels in pepper seedlings (Figure 6, *p* < 0.05). EBR treatment under NaCl stress further enhanced the accumulation of these metabolites, indicating a protective osmotic adjustment mechanism facilitated by EBR.

### 3.6. EBR Increases Glycine Betaine and ABA Levels Under Salt Stress

**Figure 7:**
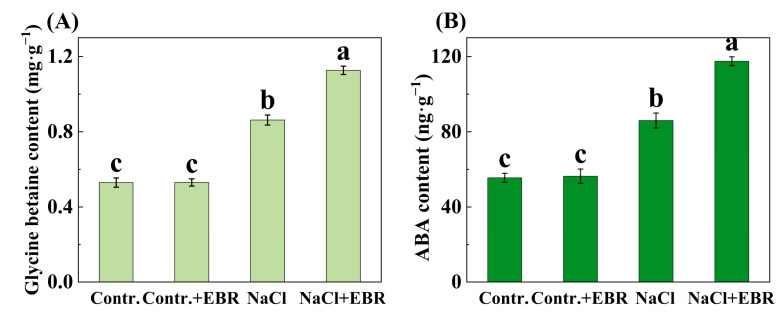
Changes in glycine betaine and ABA levels in pepper seedlings under different treatments. (**A**) Changes in Glycine betaine content. (**B**) Changes in ABA content. Different lowercase letters in the Figure indicate significant differences (*p* < 0.05, one-way ANOVA).

The levels of glycine betaine and abscisic acid (ABA) were significantly elevated under NaCl stress (Figure 7, *p* < 0.05). EBR application enhanced these levels further, indicating that EBR plays a key role in regulating osmotic stress and ABA-related signaling pathways to mitigate salt stress.

### 3.7. EBR Improves Ion Homeostasis and Reduces Salt Crystal Formation Under Salt Stress

**Figure 8:**
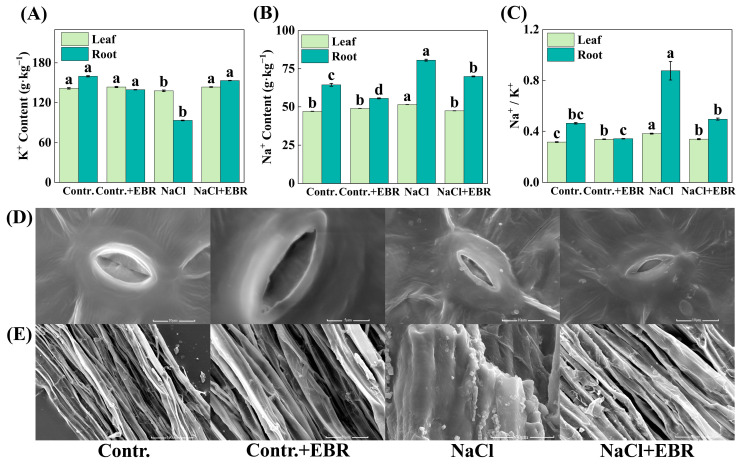
Ion content, stomatal structure, and salt crystal accumulation under different treatments. (**A**) Changes in K^+^ content. (**B**) Changes in Na^+^ content. (**C**) Changes in Na^+^/K^+^ content. Light green indicates the leaf and dark green indicates the root. (**D**) Leaf stomata imaging. (**E**) Root Duct Imaging. Different lowercase letters in the Figure indicate significant differences (*p* < 0.05, one-way ANOVA).

Under NaCl stress, EBR treatment improved ion homeostasis by increasing the K^+^ content and reducing Na^+^ accumulation in the leaves and roots. EBR also decreased the Na^+^/K^+^ ratio compared to NaCl stress alone (Figure 8). Microscopic imaging revealed that NaCl stress led to increased salt crystal formation in the leaf stomata and root ducts, while EBR application reduced salt crystal accumulation.

### 3.8. EBR Modulates the Expression of Salt-Tolerance-Related Genes in Pepper Seedlings

**Figure 9:**
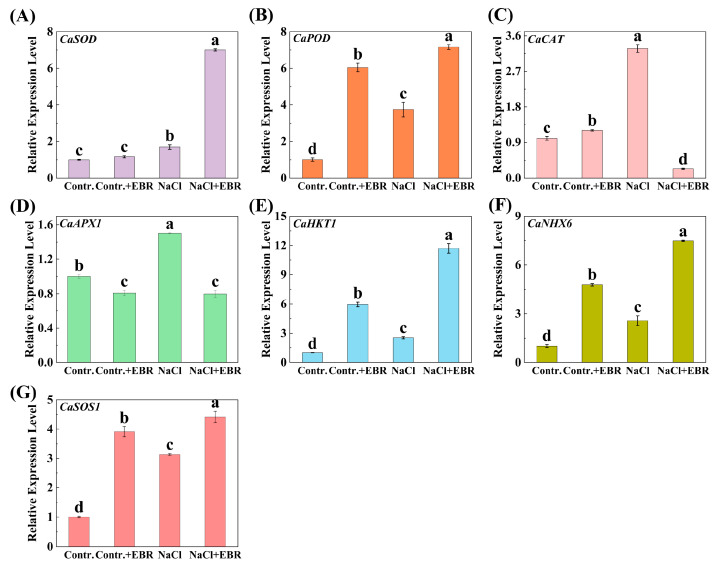
Expression levels of salt-tolerance-related genes under different treatments. (**A**) The relative expression of *CaSOD* in the four treatments. (**B**) The relative expression of *CaPOD* in the four treatments. (**C**) The relative expression of *CaCAT* in the four treatments. (**D**) The relative expression of *APX1* in the four treatments. (**E**) The relative expression of *CaHKT1* in the four treatments. (**F**) The relative expression of *CaNHX6* in the four treatments. (**G**) The relative expression of *CaSOS1* in the four treatments. Different lowercase letters in the Figure indicate significant differences (*p* < 0.05, one-way ANOVA).

A quantitative analysis showed that the expression of key genes associated with salt tolerance (*CaSOD*, *CaPOD*, *CaCAT*, *CaAPX1*, *CaHKT1*, *CaNHX6*, and *CaSOS1*) was significantly up-regulated under NaCl stress compared to the control (Figure 9). EBR treatment further increased the expression of most genes’ (*CaSOD*, *CaPOD*, *CaHKT1*, *CaNHX6*, and *CaSOS1*) expression, suggesting that EBR plays a role in improving the gene response to stress.

## 4. Discussion

In response to stress, plants typically experience a passive inhibition of growth, redirecting their internal resources to achieve a new state of homeostasis [46]. Salt stress is one of the most significant environmental factors limiting plant growth and performance, with most crops showing sensitivity to high salinity levels [47,48]. Our findings show that the foliar application of BRs (10^−7^ mol/L) significantly alleviated salt-stress-induced growth inhibition in pepper seedlings (Figure 1 and Figure 2), promoting increased plant height, stem diameter, leaf area, and biomass compared to the salt-stressed controls (Table S1). This suggests that BRs enhance pepper seedling growth and development, improving their tolerance to salt stress.

Consistent with previous studies, salt stress negatively impacted key photosynthetic parameters in pepper seedlings, but BRs treatment mitigated these effects, enhancing the photosynthetic performance (Figure 3). This suggests that BRs improve salt tolerance by regulating the stomatal aperture and enhancing carbon fixation [29,49,50]. Furthermore, BRs effectively reduced oxidative stress markers (electrolyte leakage, MDA content, H_2_O_2_, and O_2_^−^ accumulation) and ROS accumulation (Figure 4), demonstrating their antioxidant role in mitigating salt-stress-induced damage [51,52].

Our research demonstrated that salt stress affects the intracellular balance of Na^+^ and K^+^ in pepper seedlings, but BRs treatment helps regulate ion homeostasis more effectively (Figure 8). BRs promoted a Na^+^ efflux and K^+^ retention, maintaining the ion balance within the cells and mitigating salt-induced cellular damage. These results indicate that BRs treatment may improve ion homeostasis by modulating the expression of ion channels and transporter proteins, thereby enhancing the salt tolerance in pepper plants [53,54].

This study also investigated the expression levels of stress-responsive genes in pepper leaves subjected to salt stress, with or without BRs treatment. BRs treatment had a marked impact on the expression of genes (*CaSOD*, *CaPOD*, *CaCAT*, *CaAPX1*, *CaHKT1*, *CaNHX6*, and *CaSOS1*) involved in the stress response (Figure 9). These results suggest that BRs can influence the expression of stress-responsive genes, thus contributing to the tolerance of pepper plants to salt stress [55,56,57]. Concurrently, it was observed that BR treatment markedly augmented the antioxidant defense system capacity (SOD, POD, CAT, and APX) (Figure 5) and the GB and ABA content (Figure 7) in peppers. Collectively, these findings indicate that BRs may facilitate the growth and development of pepper seedlings by regulating the level of polar gene expression and antioxidant defense systems, thereby enhancing their resilience to salt stress [58,59,60].

A review of previous studies revealed that the efficiency of plant photosynthesis typically declines under salt stress, resulting in a reduction in the production of photosynthetically produced products. Plants have been observed to respond to salt stress by increasing the content of sugar, proline, and glycine betaine [20,60,61,62,63], a finding that aligns with the results of our study (Figure 6 and Figure 7A). Therefore, it is hypothesized that BR treatment activates the expression of enzymes involved in photosynthesis, promotes the synthesis of sugars, and accelerates the transport of sugars within the plant, thereby reducing the adverse effects of salt stress. Sugars not only serve as an energy source, but also influence the synthesis of sugar alcohols, which are converted into proline, glycine betaine, and other antioxidant compounds. These substances significantly enhance the plant’s stress tolerance.

Proline accumulation in response to salt stress plays a crucial role in osmoregulation, helping plant cells maintain osmotic balance. Proline also acts as an antioxidant, reacting with ROS to reduce oxidative damage to cell membranes (Figure 6C). In contrast, glycine betaine, an organic osmolyte, helps maintain an osmotic balance by reducing cellular water loss and scavenging ROS, thus mitigating salt-induced oxidative damage and preserving protein function under stress conditions. By enhancing the accumulation of these compounds, BRs improve the resilience of pepper plants to salt stress (Figure 7A).

## 5. Conclusions

This study demonstrated that BRs significantly mitigate the damage caused by salt stress in pepper plants. Under simulated salt stress, BRs-treated pepper seedlings exhibited improvements in their growth rate, photosynthetic efficiency, and oxidative stress tolerance. BRs treatment also promoted the accumulation of sugars, proline, and glycine betaine, which led to a reduction in oxidative-stress-related substances such as ROS and MDA, thereby mitigating cellular damage. Furthermore, BRs upregulated the expression of stress-responsive genes related to osmoregulation and antioxidant defenses, enhancing the plant’s ability to adapt to salt stress conditions.

## Data Availability

The original contributions presented in this study are included in the article/Supplementary Materials; further inquiries can be directed to the corresponding author.

## Supplementary Materials

The following supporting information can be downloaded at: https://www.mdpi.com/article/10.3390/plants13213029/s1, Table S1: Growth of pepper seedlings under different treatments; Table S2: Primer sequences for qRT-PCR amplification analysis.
