# Supplementary material for: Brassinosteroids Alleviate Salt Stress by Enhancing Sugar and Glycine Betaine in Pepper (Capsicum annuum L.)
Source: Plants (Basel). 2024 Oct 29;13(21):3029. doi: 10.3390/plants13213029 (PMC11548198; doi:10.3390/plants13213029)
Supplement: Supplementary file 1 [file plants-13-03029-s001.zip › plants-3254512-supplementary/Table S1.pdf]

Table S1. Growth of pepper seedlings under different treatments.

EBR (mol/L)	Stem diameter (mm)	Plant height (cm)	Fresh weight (g)	Dry weight (g)	Leaf area (cm^2)
Contr.	1.93±0.54a	6.84±0.15a	1.73±0.05a	0.17±0.016a	14.29±0.68a
NaCl	1.52±0.36b	4.64±0.30c	0.52±0.07d	0.05±0.009c	4.59±0.77c
10^{-5}	1.85±0.46a	5.54±0.14bc	0.81±0.07c	0.07±0.004c	8.69±0.72b
10^{-6}	1.76±0.73a	5.18±0.13bc	0.70±0.04cd	0.09±0.008bc	6.44±0.31c
10^{-7}	1.88±0.38a	5.78±0.86b	1.03±0.05b	0.11±0.008b	9.40±1.18b
10^{-8}	1.55±0.47b	4.80±0.37c	0.59±0.08d	0.05±0.007c	6.32±0.50c
10^{-9}	1.55±0.12b	4.78±0.33c	0.56±0.06d	0.06±0.007c	5.54±0.25c
