# Supplementary material for: Brassinosteroids Alleviate Salt Stress by Enhancing Sugar and Glycine Betaine in Pepper (Capsicum annuum L.)
Source: Plants (Basel). 2024 Oct 29;13(21):3029. doi: 10.3390/plants13213029 (PMC11548198; doi:10.3390/plants13213029)
Supplement: Supplementary file 1 [file plants-13-03029-s001.zip › plants-3254512-supplementary/Table S2.pdf]

Table S2. Primer sequences for qRT-PCR amplification analysis.

Gene		**Sequence (5'-3')**
CaHKT1	F	TCAAGCTCTATAAATTTGGAC
	R	TCACACTTCATGATTGAGTTGTAGG
CaNHX6	F	TCTACAGTCTAGTCTTCGGG
	R	GAGTTGATGTGAGAGAGGTC
CaSOS1	F	GTGCATCCTCATACTTCATCT
	R	GATTTGCTATAGTTTGCGGG
CaActin	F	AGGGATGGGTCAAAAGGATGC
	R	GAGACAACACCGCCTGAATAGC
